# A systematic review and meta-analysis assessing the impact of pentoxifylline on the healing and recurrence of venous leg ulcers

**DOI:** 10.1177/02683555241309797

**Published:** 2024-12-17

**Authors:** Marwah Salih, Hussein Elghazaly, Sarah Salih, Sarah Onida, Alun H Davies

**Affiliations:** Section of Vascular Surgery, Department of Surgery and Cancer, 4615 Imperial College London, London, UK

**Keywords:** Vascular, venous, ulcer, healing

## Abstract

**Introduction:**

Venous leg ulcers (VLU) are the most severe manifestation of venous insufficiency and carry a poor prognosis because of delayed healing and recurrent ulceration. Pentoxifylline (PTX) is an example of a vasoactive medication that can be used alongside compression therapy to help improve ulcer healing rates. A previous review highlighted improved healing of VLU with PTX, although no analysis was made for complete ulcer healing and recurrence following treatment.

**Methods:**

A systematic review was conducted according to the Preferred Reporting Items for Systematic Reviews and Meta-Analyses (PRISMA) guidelines. The EMBASE, MEDLINE and Cochrane databases were searched for all relevant English-language human studies between January 1980 and August 2023. Two independent authors screened and reviewed all articles for inclusion, performed data extraction and assessed methodological quality according to Cochrane’s risk of bias tool. Primary outcomes included complete ulcer healing and recurrence rates in the ipsilateral limb.

**Results:**

Ten studies were eligible for analysis, of which nine were randomised trials and one was an observational cohort study. There were a total of 1,025 participants, with 515 having received PTX. In those receiving 1200 mg PTX, venous leg ulcers healed in 62% (315 participants). Compared to controls, PTX administration was associated with a significantly higher likelihood of complete ulcer healing (OR 2.56, 95% CI 1.97-3.32, *p* < .001). The rate and time of ulcer recurrence were not recorded in any of the studies included.

**Conclusion:**

The evidence demonstrates that PTX may have a significant beneficial impact on the rate of complete ulcer healing. Little evidence is currently present in the literature evaluating the recurrence rates of ulcers following PTX treatment. Large scale, high quality RCTs with an adequate follow-up period are needed to evaluate this and assess whether treatment with PTX shows a significant benefit in prevention of recurrence in venous ulcers.

## Introduction

Venous leg ulcers (VLU) represent the most severe form of chronic venous insufficiency (CVI), affecting up to 2% of the population.^1–3^ VLUs are associated with delayed healing and recurrent ulceration, and this, due to their prevalence, has a significant impact on both society’s health economic burden and patients’ quality of life (QOL).^4–7^

The CEAP – Clinical, Etiological, Anatomical and Pathophysiological – classification system was initially designed to allow for the categorisation of VLUs and other clinical manifestations of venous insufficiency, an example being active venous ulcers classified as C6 within the ‘clinical’ category.^8^ The use of this system has improved the precision of pathology identification and facilitated the integration of imaging for diagnostic confirmation of CVI.^8,9^

The established first-line treatment of VLUs is compression therapy to assist with blood flow and support the veins within the affected leg. Research has demonstrated an improvement in ulcer healing in patients receiving concomitant superficial venous intervention, such as endovenous ablation, particularly in procedures carried out within 6 months of the initial occurrence of the VLU.^10^ Unfortunately, despite evidence highlighting improved patient outcomes, there remains a large proportion of patients who do not undergo the recommended intervention, likely due to inaccessibility and lack of resources. There is continued discussion on the use of pharmaceutical adjuncts that can be used alongside compression therapy.^11^ Observational studies demonstrating a link between more severe CVI and increased morbidity and mortality in patients further highlight the importance of identifying treatments that can help improve patient outcomes.^12^

Pentoxifylline (PTX), a xanthine derivative, is an example of a medication associated with increased microcirculation and anti-inflammation.^13^ PTX can be used alongside compression therapy to help improve ulcer healing rates, as demonstrated by a previous review evaluating randomised controlled trials (RCTs) by Jull et al. in 2012.^14^ Whilst this review highlights improved healing of VLU, no data was found for recurrence of venous ulcers following treatment. There has also been no updated review to assess if further evidence now exists that could support or oppose the use of PTX in the treatment of VLUs.

This systematic review and meta-analysis aims to offer an updated review of all studies assessing the effect of PTX on venous ulcer healing and recurrence, to analyse whether a statistical difference exists in healing outcomes and venous ulcer recurrence rates between patients receiving PTX compared to those who do not.

## Methods

**Figure 1.**
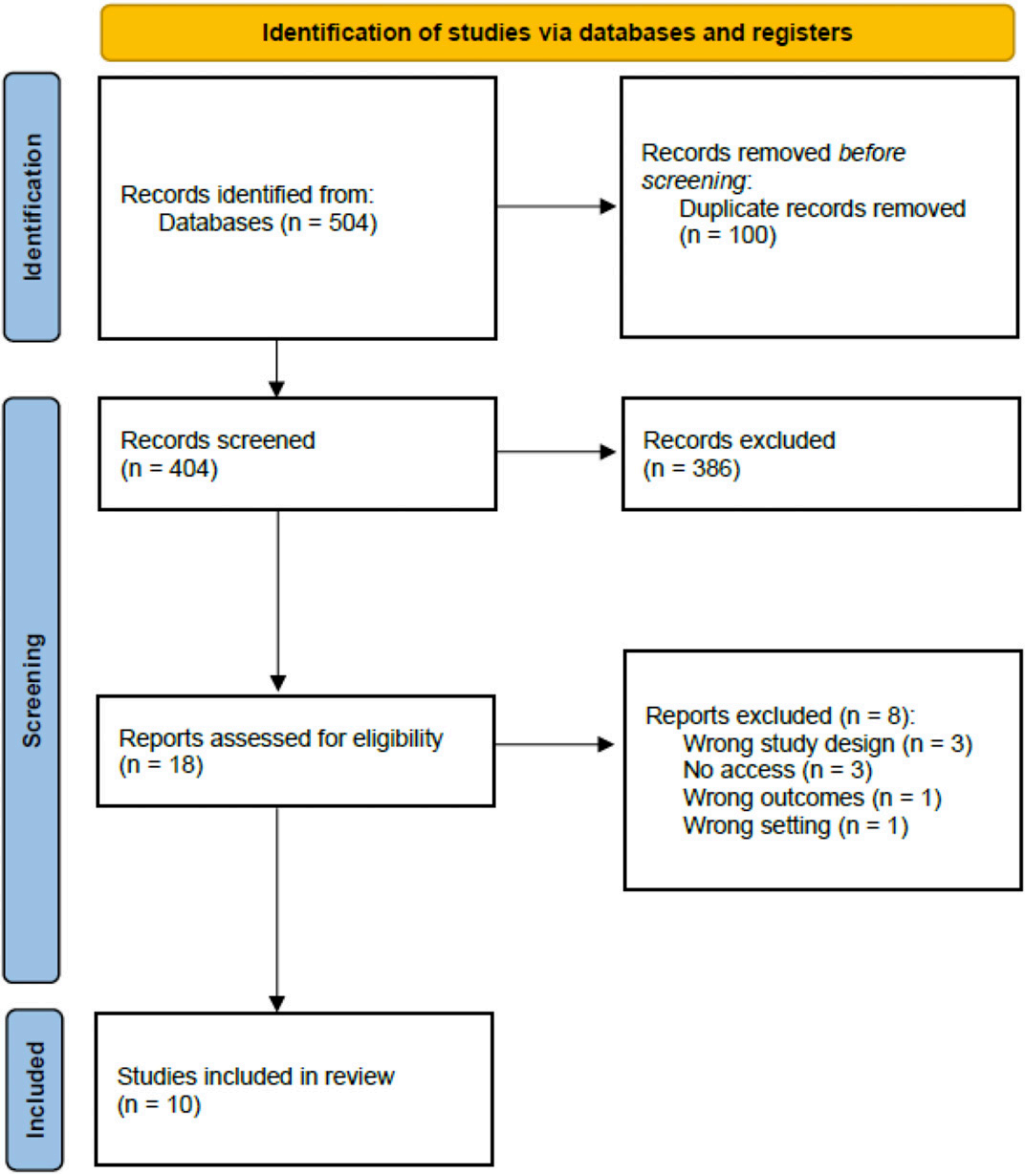
PRISMA flow diagram demonstrating the total number of studies identified, the exclusion process and the final total of papers included in this study.

A systematic review and meta-analysis was conducted in accordance with the Preferred Reporting Items for Systematic Reviews and Meta-Analyses (PRISMA) guidelines and registered on PROSPERO (CRD42024511620) (Figure 1). A systematic search was completed on several databases, including PubMed, EMBASE, Medline, CINAHL and Cochrane library, to identify all relevant articles (Supplemental Data).

### Inclusion and exclusion criteria

Inclusion criteria were any article reporting on any adult patient over 18 years of age, with a confirmed active venous leg ulcer and documented follow-up. Articles reporting on complex, mixed or arterial ulcers were excluded. Case reports, letters and opinion pieces were also excluded. The search strategy was limited to articles in the English language only.

### Data extraction and critical appraisal

All searches were exported into the reference management software programme, Covidence (Covidence systematic review software, Veritas Health Innovation, Melbourne, Australia, 2022), with all duplicates removed. Two independent reviewers completed abstract and full text reviews for inclusion and those who met the criteria were subsequently extracted. Any discrepancies in decisions were discussed with a third reviewer until consensus was reached.

Extracted data included paper characteristics, patient demographics, the number of patients with a diagnosis of CVI, the time since the current ulcer was first reported, the size/area of the active venous ulcer, the rate of complete ulcer healing, time taken for complete ulcer healing, ulcer recurrence rate and the time taken for the ulcer to recur. Where available, standard intervention given, as well as dose and duration of PTX treatment were also extracted for analysis. Means and standard deviations for each outcome were extracted.

### Statistical analysis

A random-effects meta-analysis was performed for the primary outcome of complete ulcer healing. Given that all studies had notable differences in other reported outcomes, no other meta-analysis was performed. Statistical analysis was conducted using R version 3.3.2 (R Core Team, GNU GPL v2 License) and RevMan5 (The Cochrane Collaboration, London, UK). The statistical heterogeneity of effect size among trials was quantified by using the I^2^ test considering a threshold of 50% for substantial statistical heterogeneity. A narrative synthesis was conducted where meta-analysis was not suitable.

### Quality and risk of bias assessment

The quality and certainty of the evidence included was assessed using the Grading of Recommendations, Assessment, Development and Evaluations (GRADE) Framework. Evidence was graded as high, moderate, low or very low after assessment of limitations, inconsistency, imprecision, indirectness, and publication bias within the included studies.^15^ All studies were assessed for methodological robustness using the Cochrane Risk of Bias (RoB) tool. Discrepancies were discussed with a third reviewer until consensus was reached.

## Results

The search identified 504 studies. Following the completion of the screening process, a total of ten studies were included for analysis (Table 1).^16–25^ One study that was excluded from the analysis was a trial investigating the efficacy of PTX in VLU healing with only the protocol available at the time of review.^26^

**Table 1.** Summary of the 10 papers included in this study. *RCT = randomised control trial*, *PTX = pentoxifylline*.

Author	Year	Country	Study type	Total pts	PTX group	No PTX group
Barbarino	1992	Italy	RCT	12	6	6
Belcaro	2002	Italy	RCT	172	84	88
Colgan	1990	Dublin	RCT	80	38	42
Dale	1999	Edinburgh	RCT	200	101	99
Falanga	1999	USA	RCT	129	84	45
Nelson	2007	Leeds, UK	RCT	245	121	124
Nikolovska	2002	Macedonia	RCT	80	40	40
Sanctis	2002	Italy	RCT	80	41	39
Parsa	2021	Iran	RCT	40	20	20s
Lemos	2021	Brazil	Cohort	67	-	-

### Study characteristics

Nine of the ten studies were randomised control trials, and one was an observational cohort study. There were a total of 1,025 participants, with 515 having received 1200 mg of PTX and the remaining receiving compression therapy or placebo alone. These studies took place between 1990 and 2021, with centres in the United Kingdom, Italy, Ireland, USA, Iran, Macedonia and Brazil (Table 1). Nine of the ten studies used 1200 mg PTX, divided into 400 mg three times a day, whilst one study reviewed both 1200 mg and 2400 mg PTX total dosage per day and compared this to a placebo. It was assumed that all studies used oral PTX, with only one reporting the use of IV PTX at the same total dosage per day.^16^ The majority of studies had a medication duration of 24 weeks for those receiving PTX. No studies mentioned if any superficial venous intervention, such as endovenous ablation or foam sclerotherapy, had been carried out on participants.

### Complete venous ulcer healing

**Figure 2.**
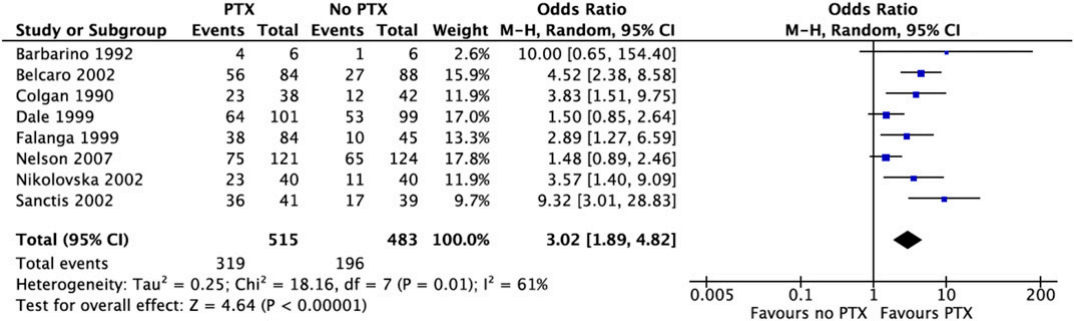
Proportion of ulcers healed in the PTX and no PTX groups. Forest plot using the random effects model demonstrating a statistically significant pooled odds ratio in favour of the pentoxifylline group (OR 3.02, 95% CI 1.89-4.82, *p* < .0001).

Within the studies included, eight randomised control trials and one cohort study assessed complete ulcer healing. Of the patients receiving PTX in the RCTs, venous leg ulcers were healed in 62% (315 out of 515 participants) after the completion of 12-24 weeks treatment with PTX. When compared to those not receiving PTX, participants within the treatment group showed a higher likelihood of complete ulcer healing (OR 3.02, 95% CI 1.89-4.82, *p* < .0001) (Figure 2). Heterogeneity between the studies was high (I^2 =^ 61%). In the cohort study, a higher trend in VLU healing was noted in 57% of patients receiving PTX, in comparison to 33% of the control group, although this was not statistically significant.

### Time to ulcer healing

The time to ulcer healing was noted in one study, with a shorter average ulcer healing time of 71 days in the higher dose PTX group receiving 800 mg three times a day, in comparison to 100 days for those receiving placebo (*p* = .04). They reported no statistical significance in the difference in complete healing between placebo and those receiving the lower dose of 400 mg three times a day of PTX.^18^

### Venous ulcer recurrence

The rate and time of ulcer recurrence were not recorded in any of the studies included.

### Adverse events

**Figure 3.**
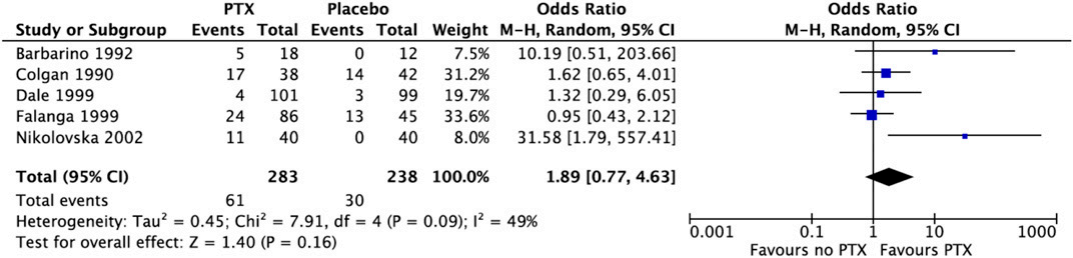
Proportion of patients with side effects was higher in the PTX group, in comparison to the group who did not receive PTX. Forest plot using the random effects model demonstrating a pooled odds ratio in favour of the pentoxifylline group (OR 1.89, 95% CI 0.77-4.63, *p* = .09).

No adverse events (AE) were recorded in the cohort study.^21^ Of the nine RCTs, five studies with a total of 521 participants reported AEs.^16,18,19,22,23^ In a random effects meta-analysis, those receiving PTX reported more AEs, although this was not statistically significant (OR 1.89, 95% CI 0.77-4.63, *p* = .09) (Figure 3). The most common AEs noted were related to the gastrointestinal system. One study reporting AEs had no withdrawals noted,^16^ whilst three studies noted participant withdrawal, with mention of AEs as a reason for this.

### GRADE assessment

The GRADE assessment was performed for the incidence of complete ulcer healing. This demonstrated evidence that was predominantly judged as moderate certainty because of the high heterogeneity in the studies.

### Risk of bias (RoB) assessment

The methodological quality of the included studies was assessed using five domains (Supplemental data, Figure 1). Overall, six RCTs were found to have some concerns.^16–20,23^ This was mainly due to lack of adherence and deviation from the published protocols. One RCT was classified as high risk of bias (ROB) within both the missing outcome domain and the measurement of outcome domain, because of the large difference between the PTX group and control group in the numbers lost to follow-up.^17^ Two RCTs had an overall low RoB.^21,22^

## Discussion

In this meta-analysis, we assessed the existing evidence in relation to the use of PTX as a pharmacological adjunct in VLUs. To the authors’ knowledge, this is the most up-to-date assessment of the available literature and included cohort studies. Our findings suggest that PTX may be a beneficial pharmacological adjunct to use alongside compression therapy in VLUs, although this conclusion is drawn from a majority of studies that were noted to have some or major concerns within their methodology. We found that, despite one of our important outcomes being the rate of ulcer recurrence, no assessment of this has been conducted in any of the existing trials. VLUs present a major economic burden and make up the majority of leg ulcers seen by the healthcare team, estimated to cost the NHS over £2 billion per annum.^27^ There is substantial evidence within the literature to suggest that, once a VLU develops, an individual is more at risk of future ulcers, thus an assessment of ulcer recurrence rates would be important in determining the true impact PTX could have on VLU outcomes.^28^ The overall health economic burden of VLUs is significant and therefore any intervention that could reduce time to ulcer healing or recurrence would have a substantial beneficial impact on the cost of care. Despite the inference that PTX might offer a cost-effective treatment due to their associated increased rate of ulcer healing, no cost-analysis was carried out in the studies included and there remains a need to identify a treatment for VLUs that is both cost-effective and allows for improved patient outcomes. Additionally, the majority of studies did not report any issues with adherence to the medication which, in the implementation of any pharmacological treatment, can be a limiting factor that must be considered. This is especially true in medication regimes that include multiple doses throughout the day over a long period of time, like that of PTX. Similar studies have been conducted to investigate adherence to micronised purified flavonoid fraction (MPFF), another venoactive drug used in VLU healing.^29^ It was noted that, when changing from a twice-daily to once-daily prescription, no change in clinical efficacy was reported and raised the possibility of improved adherence. In order to establish if PTX may be useful in this context, further trials taking this into consideration must be conducted. When conducting this literature review, a study protocol for a recently registered clinical RCT assessing the efficacy of PTX on VLU healing was noted. This study, when completed, could help to strengthen the evidence demonstrated in this review and may address the gaps within the available literature that have been highlighted from this review.^26^

The effect estimate of PTX benefit in VLU healing demonstrated high heterogeneity in this review, suggesting uncertainty in the overall findings. This may be because of the varying sample size, patient populations, and varied outcomes measured across the selected studies. However, we attempted to account for this by using a random-effects model, which considered both within-study and between-study variations, acknowledging the potential sources of diversity inherent in the pooled data. The use of the random effects model allowed us to account for unobserved factors that contribute to the observed heterogeneity, providing a more conservative and generalised estimation of the true effect size. By acknowledging and addressing the high heterogeneity through this analytical strategy, we aimed to enhance the reliability and generalisability of our findings, ensuring a more comprehensive interpretation of the evidence within the context of the research question at hand. PTX as a medication is relatively low risk, easily accessible and could be given in both primary and secondary care. This review has highlighted a lack of high quality, methodologically robust trials that could allow for a conclusion to be reached regarding the benefits on PTX in venous ulcer healing.

Interestingly, whilst there is established evidence that has demonstrated a notable benefit in venous ulcer patients who receive superficial venous intervention (e.g., endovenous ablation), there was a lack of reporting in the studies included in this review as to whether patients had received any such intervention.^10^ This highlights a further avenue of research investigating the use of PTX for VLUs in patients who have undergone superficial venous intervention, to better investigate the benefits of PTX within the patient cohort. Similar observations have been made with the use of MPFF, where including this treatment alongside surgical intervention improved patient-reported symptoms across all CEAP stages.^30^ This is especially important given current guidelines recommending superficial venous intervention in venous ulcer patients, because of demonstratable better clinical outcomes. A recent trial protocol that was excluded from our review did not mention accounting for patients who have undergone superficial venous interventions specifically.^26^ This would be an important factor to note to ensure that the efficacy of PTX on VLU healing can be accurately assessed in both the patients who have undergone superficial venous intervention and those who have not, given this could impact the overall outcomes noted.^26^

### Study limitations

A key limitation of this meta-analysis stems from the temporal scope of the available research in this field. It must be noted that the majority of papers assessing PTX were performed at a time when study methodology and reporting standard were not as robust as they are today. Because of this, the majority of studies with small sample sizes, lack of documentation of power calculations, variable follow-up and reporting outcomes must all be considered when drawing conclusions from the evidence. Further to this, the GRADE assessment of certainty of the evidence for ulcer healing rate was moderate, suggesting that the results may have been underpowered.

## Conclusion

In conclusion, this review has highlighted the potential benefit in the use of PTX as a pharmacological adjunct alongside compression therapy for VLUs. Given that the majority of evidence suggesting this to be the case was conducted at a time when trial reporting of results underwent a less regimented peer-reviewed system, there is a need for a more robust, large-scale RCT to be carried out to confirm the results demonstrated within this review and also to evaluate if there is an adjuvant benefit of PTX in patients receiving endovenous treatment.

## Supplemental Material

Supplemental Material - A systematic review and meta-analysis assessing the impact of pentoxifylline on the healing and recurrence of venous leg ulcers Supplemental Material for A systematic review and meta-analysis assessing the impact of pentoxifylline on the healing and recurrence of venous leg ulcers in Phlebology.
