# Supplementary material for: A systematic review and meta-analysis assessing the impact of pentoxifylline on the healing and recurrence of venous leg ulcers
Source: Phlebology. 2024 Dec 17;40(6):379–85. doi: 10.1177/02683555241309797 (PMC12188020; doi:10.1177/02683555241309797)
Supplement: Supplemental Material - A systematic review and meta-analysis assessing the impact of pentoxifylline on the healing and recurrence of venous leg ulcers [file sj-pdf-1-phl-10.1177_02683555241309797.pdf]

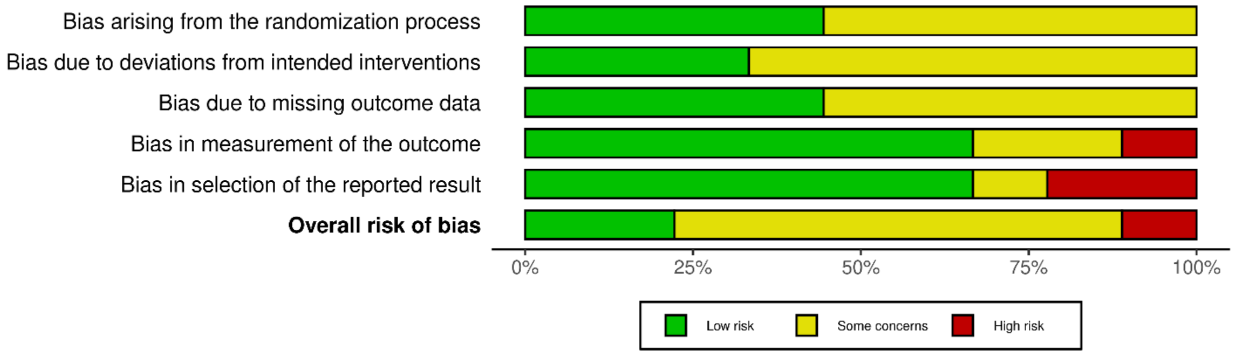

S Figure 1 – Risk of bias analysis of the papers included in this study. There was an overall moderate-high risk of bias noted in the studies included.
